# The High-Pressure Freezing Laboratory for Macromolecular Crystallography (HPMX), an ancillary tool for the macromolecular crystallography beamlines at the ESRF

**DOI:** 10.1107/S2059798323010707

**Published:** 2024-01-24

**Authors:** Philippe Carpentier, Peter van der Linden, Christoph Mueller-Dieckmann

**Affiliations:** a Université Grenoble Alpes CEA CNRS, IRIG–LCBM UMR 5249, 17 Avenue des Martyrs, 38000 Grenoble, France; b ESRF, The European Synchrotron, 71 Avenue des Martyrs, 38000 Grenoble, France; c ESRF, PSCM (Partnership for Soft Condensed Matter), 71 Avenue des Martyrs, 38000 Grenoble, France; STFC Rutherford Appleton Laboratory, United Kingdom

**Keywords:** high pressure, macromolecular crystals, gas derivatives, protein channels, HPMX, High-Pressure Freezing Laboratory for Macromolecular Crystallography, ESRF

## Abstract

This article describes the High-Pressure Freezing Laboratory for Macromolecular Crystallography (HPMX) at the ESRF, and highlights new and complementary research opportunities that can be explored using this facility. The laboratory is dedicated to investigating interactions between macromolecules and gases *in crystallo*, and finds applications in many fields of research, including fundamental biology, biochemistry, and environmental and medical science. At present, the HPMX laboratory offers the use of different high-pressure cells adapted for helium, argon, krypton, xenon, nitrogen, oxygen, carbon dioxide and methane. Important scientific applications of high pressure to macromolecules at the HPMX include noble-gas derivatization of crystals to detect and map the internal architecture of proteins (pockets, tunnels and channels) that allows the storage and diffusion of ligands or substrates/products, the investigation of the catalytic mechanisms of gas-employing enzymes (using oxygen, carbon dioxide or methane as substrates) to possibly decipher intermediates, and studies of the conformational fluctuations or structure modifications that are necessary for proteins to function. Additionally, cryo-cooling protein crystals under high pressure (helium or argon at 2000 bar) enables the addition of cryo-protectant to be avoided and noble gases can be employed to produce derivatives for structure resolution. The high-pressure systems are designed to process crystals along a well defined pathway in the phase diagram (pressure–temperature) of the gas to cryo-cool the samples according to the three-step ‘soak-and-freeze method’. Firstly, crystals are soaked in a pressurized pure gas atmosphere (at 294 K) to introduce the gas and facilitate its inter­actions within the macromolecules. Samples are then flash-cooled (at 100 K) while still under pressure to cryo-trap macromolecule–gas complexation states or pressure-induced protein modifications. Finally, the samples are recovered after depressurization at cryo-temperatures. The final section of this publication presents a selection of different typical high-pressure experiments carried out at the HPMX, showing that this technique has already answered a wide range of scientific questions. It is shown that the use of different gases and pressure conditions can be used to probe various effects, such as mapping the functional internal architectures of enzymes (tunnels in the haloalkane dehalogenase DhaA) and allosteric sites on membrane-protein surfaces, the interaction of non-inert gases with proteins (oxygen in the hydrogenase ReMBH) and pressure-induced structural changes of proteins (tetramer dissociation in urate oxidase). The technique is versatile and the provision of pressure cells and their application at the HPMX is gradually being extended to address new scientific questions.

## Abstract 2

The High-Pressure Freezing Laboratory for Macromolecular Crystallography (HPMX) at the ESRF allows the preparation of gas derivatives of macromolecular crystals suitable for X-ray diffraction data collection on macromolecular crystallography beamlines. Information obtained from pressurized crystals and/or gas-derivatized structures enables the improved understanding of specific issues in structural biology, such as the internal functional architecture of proteins, the interactions and reactivity of gases with macromolecules and functional structural changes including ligand-binding processes.

## 1. Introduction

**Figure 1:**
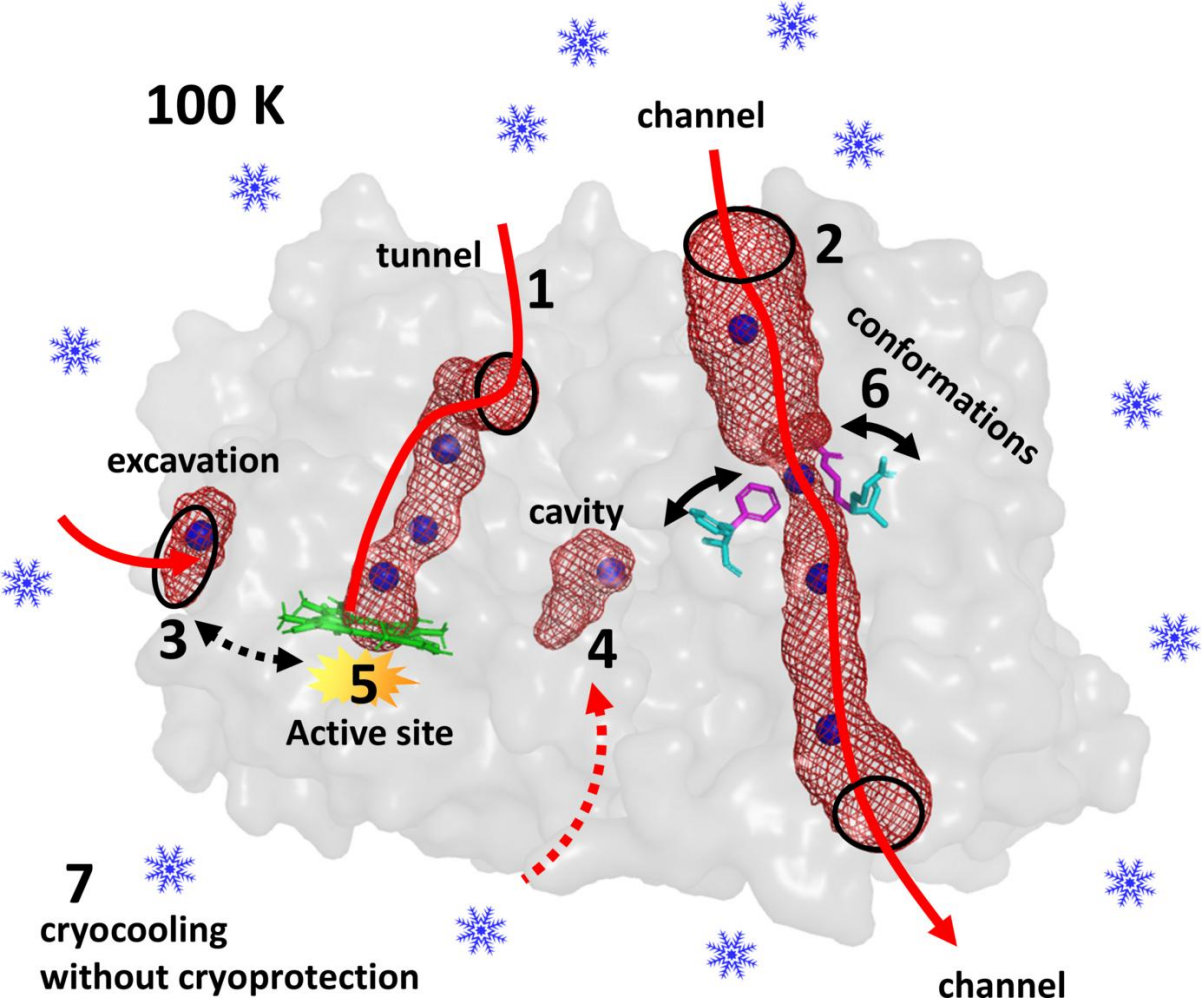
Different applications of pressurized gases in macromolecular crystallography. (1) Mapping of tunnels connecting the external solvent to the buried active site of an enzyme, (2) of transport channels passing through macromolecules, (3) of excavations at surfaces of macromolecules that may have allosteric effects (3↔5) and (4) of internal cavities. (5) Studies of possible reactions of non-inert gases (oxygen, carbon dioxide and methane…) at the active sites of specific enzymes. (6) Exploration of protein conformational fluctuations induced by pressure. (7) Cryo-cooling of crystals without any addition of cryo-protectant at 2000 bar.

The High-Pressure Freezing Laboratory for Macromolecular Crystallography (HPMX) is a complementary service of the Structural Biology (SB) Group at the European Synchrotron Radiation Facility (ESRF), providing additional and complementary functionality to the diffraction, scattering and imaging beamlines of the SB Group. This platform is dedicated to the preparation of gas derivatives of macromolecular crystals and to the investigation of interactions between macromolecules and gases *in crystallo*. The use of high pressure in macromolecular crystallography (MX) started in 2014 at the ESRF with a high-pressure cooling setup aimed at flash-cooling crystals without adding cryo-protection using helium gas at 2000 bar (van der Linden *et al.*, 2014 ▸). The system was made routinely available to the ESRF international community in 2015. This method, *a priori* specifically dedicated to a single task, paved the way for a broader range of applications when considering other gases. Indeed, a simple search of the Protein Data Bank (PDB) reveals that there are, to date, more than 1500 deposited structures containing one of the gas atoms or molecules most commonly observed in biology (Table 1 ▸). These gases are mainly involved in the function of these proteins or show essential biological properties (Table 1 ▸). While some proteins crystallize with the gas naturally present in their structures, in many others the gas has to be introduced by pressure. This observation shows that there is a clear underestimation of the presence of gases in macromolecules and a strong interest in developing specific high-pressure tools for studying protein–gas interactions (Lafumat *et al.*, 2016 ▸). The scheme in Fig. 1 ▸ summarizes the different types of application of pressurized gases that are currently available to ESRF users to complement their biological studies. A common application is the use of pressurized noble gases (argon, krypton or xenon) as labels to map the architecture of functional voids in proteins [features 1–4 in Fig. 1 ▸; tunnels (1), channels (2), excavations (3) and cavities (4)]. While cavities, channels and tunnels can also be predicted using programs, high pressure is the method of choice to visualize the routes of gas diffusion in proteins experimentally. Noble-gas labelling can also serve to solve the phase problem in MX. Pressurized non-inert gases such as oxygen, carbon dioxide and methane can be used to study their reactivity with enzymes whose function depends on these gases (feature 5 in Fig. 1 ▸). Alongside this, photolabile caged compounds have recently been used to release gas molecules into crystals in a time-controlled manner for time-resolved studies of specific enzymatic reactions (Tosha *et al.*, 2017 ▸). High helium pressure can additionally be used to probe the dynamics of proteins in crystals (feature 6 in Fig. 1 ▸) or to cryo-cool samples without adding cryo-protectant (feature 7 in Fig. 1 ▸). The aim of this article is to present the range of services offered at the ESRF HPMX laboratory to complement diffraction data collection on MX beamlines, to provide potential users with an overview of the different types of pressurized gas applications, and to describe the instruments and methods that are available to perform these high-pressure experiments. Some of these applications have already produced published scientific results.

## 2. Methods and instrumentation

### 2.1. The soak–freeze method

The ‘soaking method’ is the most common procedure used to prepare crystals of protein–ligand complexes. In practice, native protein crystals are soaked in their crystallization conditions with the mother liquor enriched with a highly concentrated solution of ligand molecules, which are thus driven to populate the protein sites of affinity. The experimental procedure becomes more complicated and requires the use of specific instrumentation when it comes to ‘derivatizing’ macromolecules with gases (*i.e.* the use of pressure cells and handling tools), although the basic ‘soaking’ concept is the same. It is indeed relatively difficult to dissolve gases in the crystal mother liquor (for example by bubbling) since their solubility is usually low at atmospheric pressure (see Table 2 ▸) and their volatility is high. Since gas solubility increases with pressure (Table 2 ▸), the common method used to efficiently label proteins with a gas by soaking is to pressurize crystals with their mother liquor (to prevent dehydration) inside specific pressure cells.

**Figure 2:**
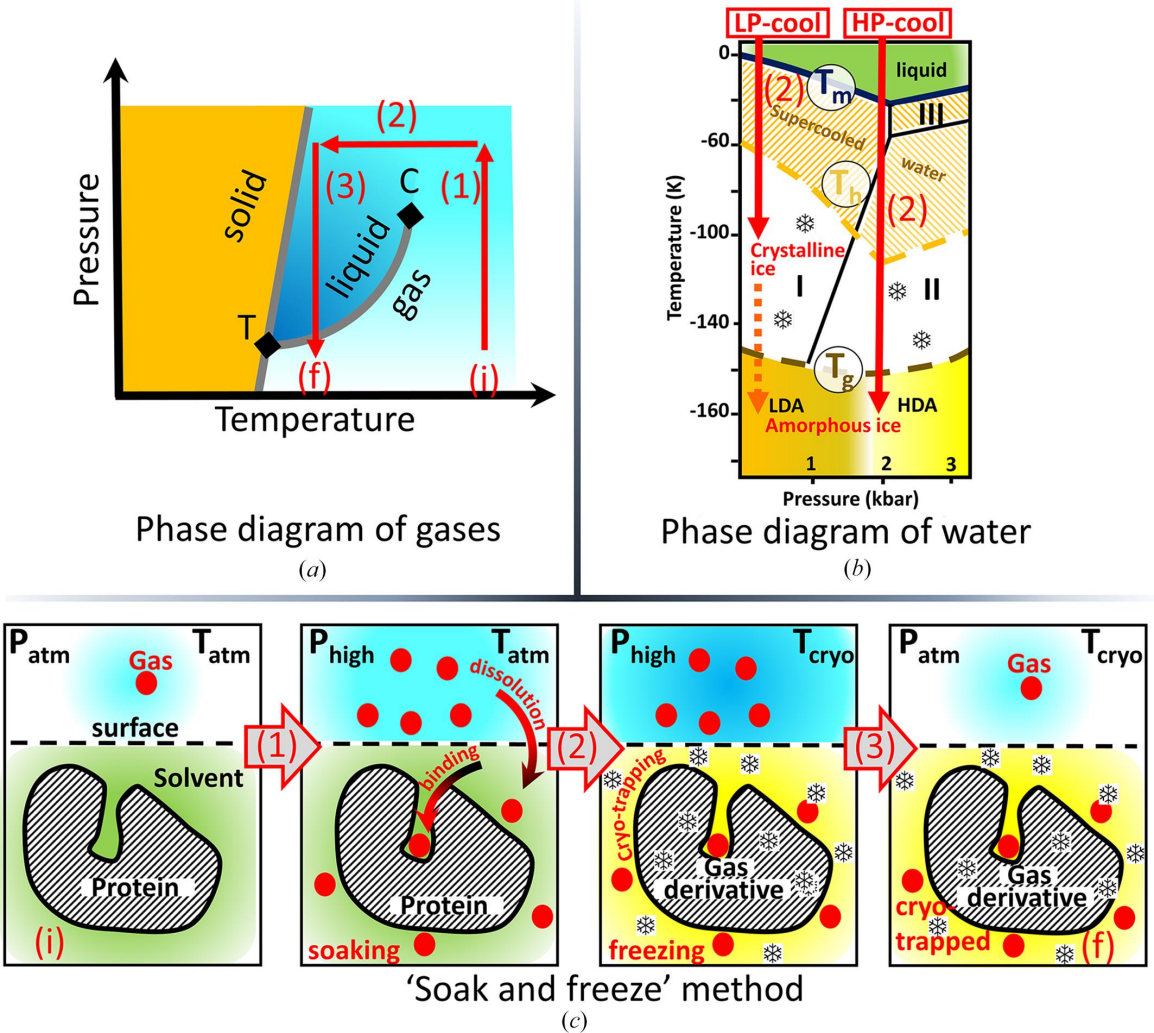
Processing of protein crystals in phase diagrams of water and gases. (*a*) Typical schematic phase diagram for helium, argon, krypton, xenon, oxygen, carbon dioxide and methane. The gas, liquid and solid phases are coloured cyan, blue and orange, respectively; the transition lines are in grey. C and T are the critical and triple points, respectively. The thermodynamic pathway of the ‘soak-and-freeze’ method used to process the protein crystals in pressure cells is shown in red: (i) initial state, (1) isothermal pressurization, (2) isobaric flash-cooling, (3) isothermal depressurization, (f) final state. (*b*) Phase diagram of water. At atmospheric pressure (LP-cool) flash-cooling of solvent produces type I crystalline ice (hexagonal), while at high pressure (HP-cool) it produces amorphous high-density ice (HDA) [avoiding crystalline ice type II (rhombohedral)]. (*c*) The different stages of the ‘soak-and-freeze’ methodology, which enables the production and cryo-trapping of gas derivatives of macromolecular crystals under pressure.

Historically, many pressure-cell systems (original and commercial) have been designed for this purpose. The first were room-temperature cells, in which the crystals are mounted in a sealed capillary and continuously pressurized during data collection. In theory, this allows the use of many gas types, but with the limitations of radiation damage, of the complexity of the pressure system to be installed on the beamline and of the hazards associated with the use of most gases under pressure (Tilton, 1988 ▸; Schiltz *et al.*, 1994 ▸; Stowell *et al.*, 1996 ▸; Quillin *et al.*, 2000 ▸; Colloc’h *et al.*, 2007 ▸). Cryogenic cells were then designed in which the derivatives are formed under pressure, and only after depressurization are the samples plunged into a cryogen to cryo-trap the gas–protein complexes. The strongest limitation of this method is that most gases escape the crystal between the depressurization and cooling steps (Sauer *et al.*, 1997 ▸; Soltis *et al.*, 1997 ▸; Djinovic-Carugo *et al.*, 1998 ▸; Vernède & Fontecilla-Camps, 1999 ▸; Vojtěchovský *et al.*, 1999 ▸; Hayakawa *et al.*, 2008 ▸). Finally, pressure cells have been designed to flash-cool samples under xenon pressure (Rigaku MSC Cryo-Xe-Siter), but using non­standard instrumentation and without taking into account the inevitable gas icing (Mizuno *et al.*, 2013 ▸). Indeed, the phase diagram (Fig. 2 ▸ *a*) shows that all gases of interest transit to a solid phase at cryogenic temperatures (77–160 K) and therefore cryogenic pressure cells require a design that regulates both pressure and temperature to properly cryo-cool samples under pressure while avoiding the gas-to-ice transformation. The ‘soak-and-freeze’ method (shown schematically in Fig. 2 ▸) was specially developed to introduce various types of gas into protein crystals, which are cryo-cooled under pressure taking into account their phase diagrams (Lafumat *et al.*, 2016 ▸). The three steps of the thermodynamic pathway with which macromolecular crystals are processed in a ‘pressure–temperature’ phase diagram of the gases are detailed in Fig. 2 ▸(*a*). The transformation of a single macromolecule within a crystal with its surrounding solvent during the ‘soak-and-freeze’ thermodynamic treatment is depicted in Fig. 2 ▸(*c*). Initially (i) the crystal is kept under ambient conditions (*T*_atm_, *P*_atm_). During step (1) (the soaking phase) the sample undergoes an isothermal pressurization in a pure atmosphere of the gas of interest that, in turn, dissolves in the solvent owing to the pressure applied according to Henri’s law (see the pressure-dependent solubility constants in Table 2 ▸). The highly concentrated gas in the solvent then diffuses rapidly and permeates the crystal to subsequently interact with the macromolecules, while at the same time populating specific binding sites (Fig. 2 ▸ *c*). During step (2) (the cryo-cooling phase) the crystal undergoes isobaric flash-cooling to cryo-trap the protein–gas complexation state formed in the previous step. The flash-cooling temperature is regulated above that of the gas-to-ice transformation to avoid cell obstruction (Table 2 ▸, Fig. 2 ▸). In step (3) (the recovery phase) the sample undergoes an isothermal depressurization at cryogenic temperatures, at which the complexation state (f) remains stable as long as the cold chain is not interrupted. The pressurization range of each gas depends on (i) the thermodynamic limit to avoid the gas-to-ice transition, (ii) the capabilities of the equipment to compress each gas and, above all, (iii) the scientific application. For most applications, moderate pressures are sufficient (0–500 bar; see Table 2 ▸ for the maximum practical values for each gas). Pressure levels and soaking times can also be used to identify low-affinity binding sites by cryo-cooling samples at different increasing values of these two parameters: secondary sites are progressively populated and appear gradually in electron-density maps (Kalms *et al.*, 2018 ▸; Ardiccioni *et al.*, 2019 ▸). However, the pressures are limited by the thermophysical properties of the gas and the technology, while soaking times need to take the dehydration of samples into consideration. For noble gases, binding sites with low occupancies are confidently identified using their anomalous scattering contribution (see Section 3.1). Finally, the presence of molecular gases (oxygen, carbon dioxide and methane) in protein crystals, even at low occupancies, can be confirmed by *in crystallo* Raman spectroscopy owing to their typical vibrational frequencies (Katona *et al.*, 2007 ▸; von Stetten *et al.*, 2015 ▸; Carpentier *et al.*, 2007 ▸).

In complement, Fig. 2 ▸(*b*) shows an interesting property in the pressure–temperature phase diagram of water that allows the preservation of crystal quality upon flash-cooling. At low pressure (for example at ambient pressure) the formation of water ice can only be avoided by adding cryo-protection prior to flash-cooling the sample to preserve the diffraction power (LP-cool path in Fig. 2 ▸ *b*). In contrast, when the solvent is flash-cooled under a high pressure of helium or argon (near 2000 bar), water is directly transformed into high-density amorphous ice without the addition of cryo-protectant to the solution (HP-cool path in Fig. 2 ▸ *b*). The amorphous matrix produced during this treatment has been described to better preserve the diffraction quality of macromolecular crystals (resolution and mosaicity; Kim *et al.*, 2005 ▸; van der Linden *et al.*, 2014 ▸).

### 2.2. The HPMX laboratory, the cryogenic pressure cells and practical methods

**Figure 3:**
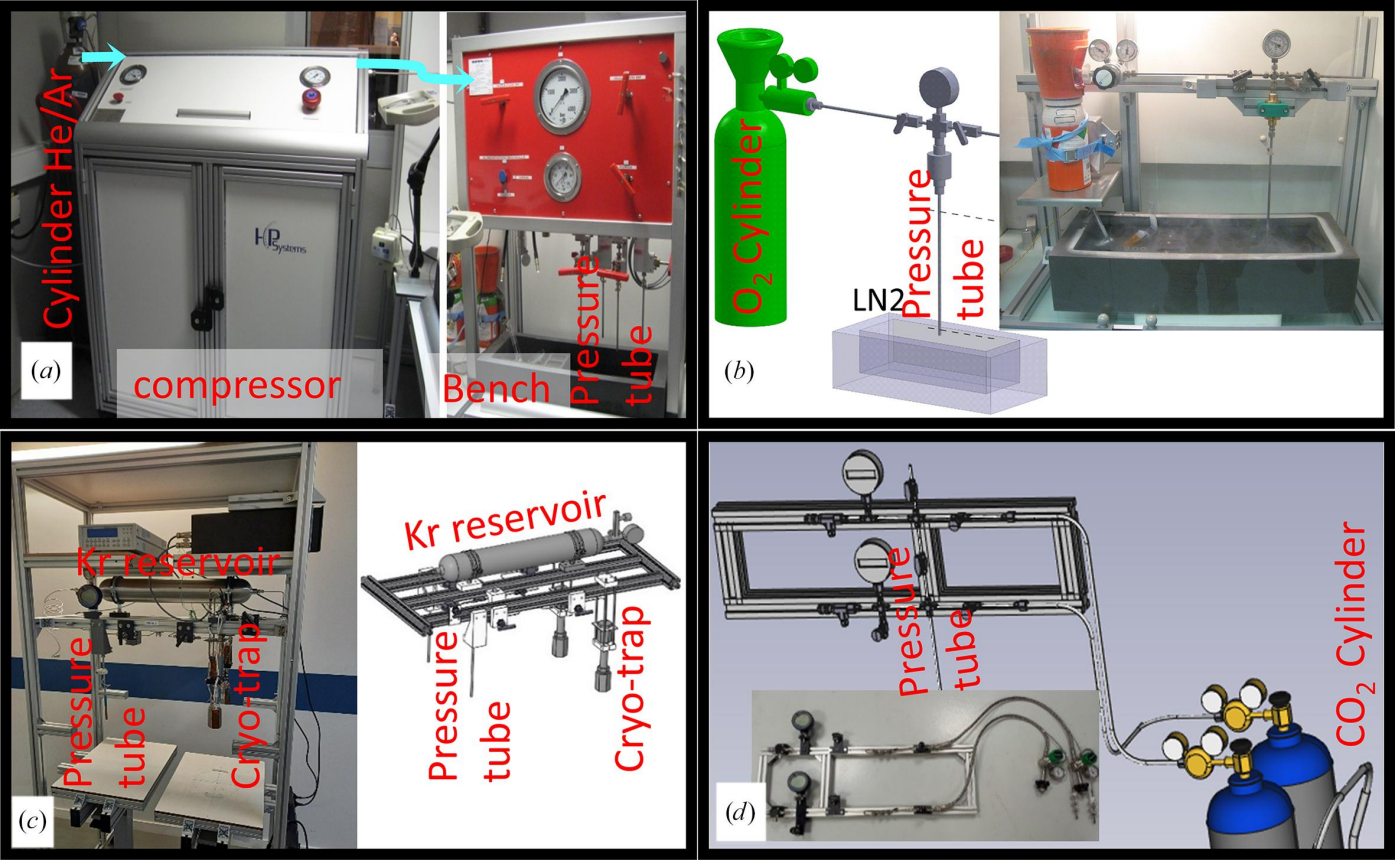
Photographs of cryogenic high-pressure cells in the HPMX. (*a*)–(*d*) Cryogenic high-pressure setups for helium/argon, molecular oxygen, krypton and carbon dioxide, respectively. The design of the methane pressure cell, which is not shown in the figure, is similar to that for oxygen.

The High-Pressure Freezing Laboratory for Macromolecular Crystallography (HPMX) is located in the centre of the structural biology village in Sector 30 (Theveneau *et al.*, 2013 ▸) that includes the automatic and mini-beam beamlines MASSIF-1 (ID30A-1) and MASSIF-3 (ID30A-3) (von Stetten *et al.*, 2020 ▸; Mueller-Dieckmann *et al.*, 2015 ▸; Bowler *et al.*, 2015 ▸), the energy-tuneable beamline ID30B (McCarthy *et al.*, 2018 ▸), the bioSAXS beamline BM29 (Tully *et al.*, 2023 ▸), the time-resolved serial synchrotron crystallography (TR-SSX) beamline ID29 and the *in crystallo* Optical Spectroscopy (*ic*OS) laboratory (von Stetten *et al.*, 2015 ▸). The HPMX is a sample-preparation laboratory which gives users access to a sample-handling bench with specific tools and equipment, a fume hood for handling hazardous samples and gases (for example oxygen), an anoxic glove bag for oxygen-sensitive proteins and, importantly, the different high-pressure cells shown in Fig. 3 ▸ (Lafumat *et al.*, 2016 ▸; van der Linden *et al.*, 2014 ▸, 2022 ▸). Each of the high-pressure cells was specifically designed taking the thermo-physical properties of the gas that it uses into account (Table 2 ▸) in order to prepare gas derivatives using the ‘soak-and-freeze’ method (Section 2.1).

**Figure 4:**
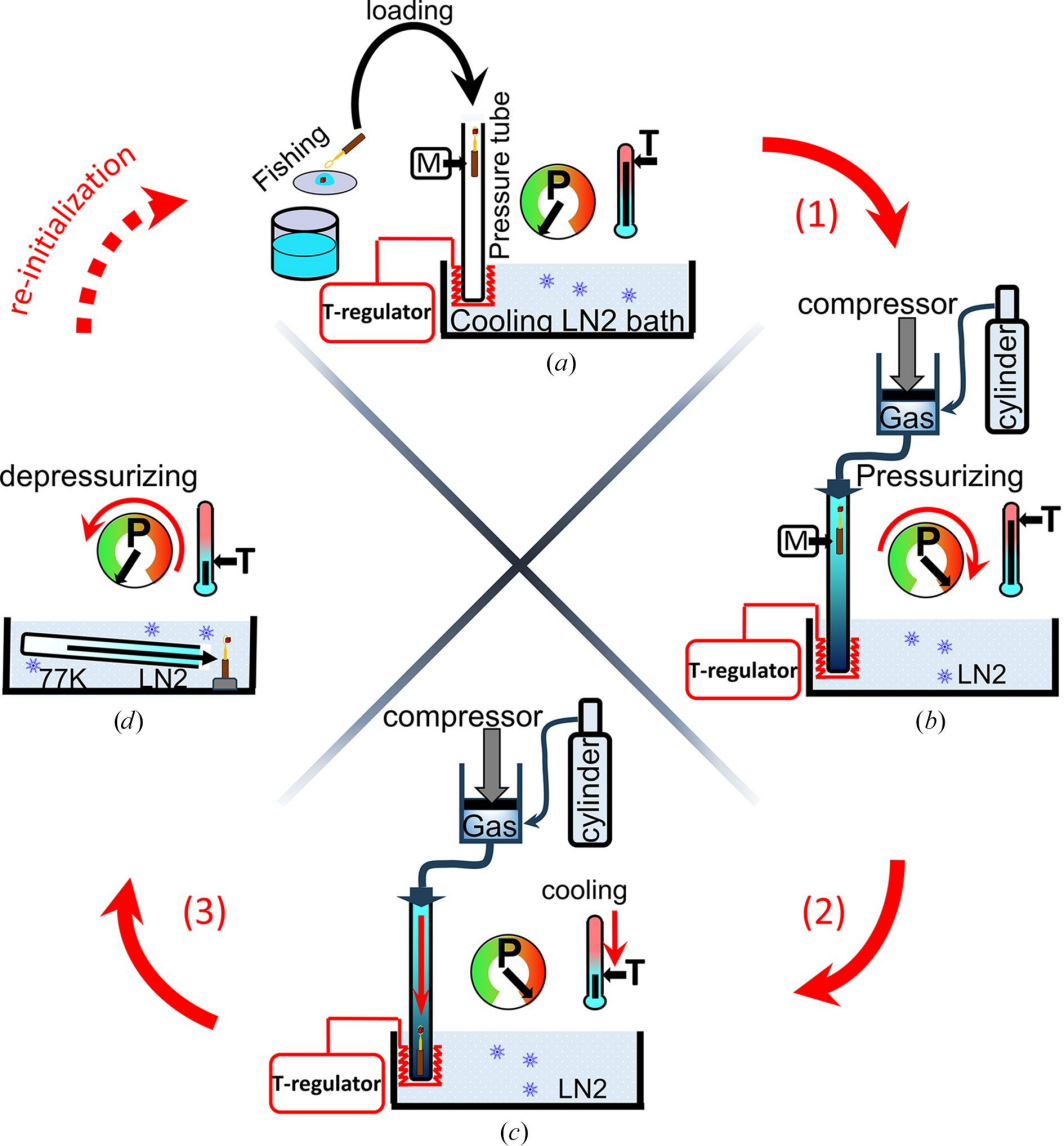
High-pressure cooling process. (*a*) The macromolecular crystal is harvested from a crystallization tray and loaded into the pressure tube at ambient pressure and temperature. The pin with the crystal is held in the upper part of the pressure tube by a strong magnet (M). (*b*) The crystal is pressurized in the tube at ambient temperature. (*c*) The crystal is cryo-cooled in the tube under pressure by releasing it from the upper part and dropping it abruptly into the lower part of the tube, which is maintained at a regulated cryogenic temperature. (*d*) The crystal is recovered manually from the tube at ambient pressure within the cryogenic bath. The red arrows indicate the three steps of the high-pressure cooling thermodynamic pathway (from Fig. 2 ▸).

The basic design of the different high-pressure cells, which is shown in Fig. 3 ▸ and schematically in Fig. 4 ▸, is almost identical. The system comprises a vertically oriented pressure tube in which the crystal inside a sample holder is loaded, a cylinder to supply the selected gas and, if needed, a system of compression to increase the pressure at the sample above that of the cylinder. The lower extremity of the pressure tube is regulated at a well defined cryogenic temperatures at which the gas remains fluid (with a temperature regulator plunged into liquid nitrogen) to cryo-cool the sample once it has been released from its initial position at the upper extremity. In practice, users bring their protein crystals to the HPMX laboratory in crystallization trays. Crystals, together with a small quantity of mother liquor, are harvested directly from the crystallization drops and mounted inside an X-ray-transparent capillary glued at the extremity of a specific high-pressure pin that has previously been described (Lafumat *et al.*, 2016 ▸; van der Linden *et al.*, 2014 ▸) and is commercially available from MiTeGen (Ithaca, New York, USA). The small amount of mother liquor inside the capillary avoids dehydration of the protein crystal during the typical 5 min of handling and should contain cryo-protectant when using low to medium pressures (below 1500 bar). In order to fish the samples out easily using this type of support, it is preferable to use crystals larger than 50 µm along at least one of the directions, although we have already been capable of successfully mounting microcrystals that were originally grown for serial crystallography (∼10 µm). The sample is loaded at the top of a pressure tube, where it is maintained at ambient temperature and pressure by means of a strong magnet, while the bottom extremity of the tube, as described above, is at cryogenic temperature (in liquid nitrogen; Fig. 4 ▸ *a*). In the next step, the tube is pressurized by the gas of interest such that the sample, still at room temperature, is exposed to high pressure, producing the protein–gas complex (the soaking step; Fig. 4 ▸ *b*). After typically 5 min of soaking in the pure pressurized atmosphere, the sample is released and drops by gravity into the lower part of the tube (by removing the holding magnet), where it is rapidly cooled to cryogenic temperature while still at high pressure to cryo-trap the protein–gas complex (the cryo-cooling step; Fig. 4 ▸ *c*). There is very little information in the literature about the influence of crystal soaking time in gases on the formation of effective derivatives (Schiltz *et al.*, 2003 ▸). In our experience, the diffusion of light gas atoms in a crystal to bind to protein sites occurs on a timescale from few seconds to minutes. The result depends on the solvent composition, the size of the crystals and the accessibility and the affinity of the gases to the binding sites. To take this aspect into account, we can cryo-cool derivatives after soaking times ranging from a few seconds to one hour, with the limitations of handling time and maintaining sample hydration under pressure (Kalms *et al.*, 2018 ▸). Finally, after cryo-cooling, the pressure is released and the tube is disconnected from the system and toppled down in the cryogenic bath, where the sample is manually extracted at cryogenic temperature and ambient pressure (the extraction step; Fig. 4 ▸ *d*). The cryo-cooled crystals, mounted on specific supports, are handled and kept at liquid-nitrogen temperatures to preserve the protein–gas complexation state. These crystals are then transferred into EMBL/ESRF-SC3 pucks for sample changers (Cipriani *et al.*, 2006 ▸) ready for diffraction data collection on the ESRF MX beamlines.

## 3. Applications of high pressure

### 3.1. Noble-gas derivatization of protein crystals and mapping of tunnels, channels and pockets

The primary application for the use of pressurized gases is the mapping of surfaces and internal structural voids present in proteins (Carpentier *et al.*, 2020 ▸, 2022 ▸; Engilberge *et al.*, 2020 ▸; Markova *et al.*, 2020 ▸; Colloc’h *et al.*, 2017 ▸; Kalms *et al.*, 2016 ▸; Ardiccioni *et al.*, 2019 ▸; Zacarias *et al.*, 2020 ▸; Melnikov *et al.*, 2022 ▸; Montet *et al.*, 1997 ▸). These empty volumes play crucial roles in conformational flexibility and in molecular mechanisms that allow biomacromolecules to fulfil their function. These structural voids can be tunnels that connect the surface of the enzyme to buried active sites (feature 1 in Fig. 1 ▸), thus selecting substrates and allowing them to diffuse to the reactive centre, while products are evacuated by the same or possibly other routes (Prokop *et al.*, 2012 ▸). Voids can also be channels passing through macromolecules that serve as an exclusive passage for small molecules (ions, water molecules *etc.*), such as in transport membrane proteins (feature 2 in Fig. 1 ▸). Macromolecules can also expose functional voids on their surfaces, such as pockets or grooves (feature 3 in Fig. 1 ▸) that may bind ligands with potentially allosteric effects or that may host structuring molecules such as lipids for membrane proteins. Finally, some macromolecules enclose cavities that transiently store small molecules (substrates, ligands, cofactors *etc.*) or accommodate them inside reactive environments (an active site) to perform reactions (features 3, 4 and 5 in Fig. 1 ▸). The architectures made by voids are difficult to visualize inside protein structures. Some of them can be detected *in silico* using various software packages, such as *CASTp* (Dundas *et al.*, 2006 ▸) and *Fpocket* (Le Guilloux *et al.*, 2009 ▸), which allow surface-pocket detection, *SURFNET* (Laskowski, 1995 ▸) and *Q-SiteFinder* (Laurie & Jackson, 2005 ▸), which predict ligand-binding sites, and *Caver* (Chovancova *et al.*, 2012 ▸) and *MOLE* (Sehnal *et al.*, 2013 ▸), which identify channels and tunnels. From an experimental standpoint, noble gases (such as argon, krypton and xenon) are commonly used to map structural voids (Kalms *et al.*, 2016 ▸; Colloc’h *et al.*, 2017 ▸; Zacarias *et al.*, 2020 ▸; Montet *et al.*, 1997 ▸). These inert gas atoms bind to hydrophobic patches of macromolecules *via* weak induced-dipole/induced-dipole interactions (London forces), but also bind in polar environments via permanent-dipole/induced-dipole interactions (Debye forces) (Schiltz *et al.*, 2003 ▸). Hence, the binding strengths and occupancies of noble gases in protein pockets increase with their polarizability (α_argon_ < α_krypton_ < α_xenon_) and their concentration in the solvent, which can be modulated by the applied pressure on the system (Table 2 ▸). With lighter gases being more volatile, one can state as a rule of thumb that xenon derivatives should be prepared at pressures of around 10 bar, those using krypton at around 100 bar and those using argon near 1000 bar. Additionally, the different radii of noble-gas atoms (*R*_argon_ < *R*_krypton_ < *R*_xenon_) may help in probing protein voids of various sizes (Table 2 ▸).

Derivatization of macromolecules using argon, krypton or xenon has been used in the past to solve the phase problem in *de novo* structure prediction using anomalous diffraction methods (SAD or MAD; Schiltz *et al.*, 2003 ▸). Since noble gases bind to proteins reversibly via weak forces, the gas derivatives are generally highly isomorphous with native crystals (without gas treatment) when the gas is introduced at low and medium gas pressures (below 500 bar). At higher pressures the gas medium can induce significant structural changes and break this isomorphism (see Section 3.3). Table 2 ▸ shows that at an energy of 6 keV, the anomalous scattering factor *f*′′ of xenon reaches 11.6 e^−^, making it easily measurable in an X-ray diffraction experiment, while that of argon is as low as 1.5 e^−^, making *de novo* phasing possible but more complicated. Furthermore, the *K* absorption edge of krypton (14.3 keV) falls within the reachable energy range of MX beamlines, allowing it to be confidently identified using anomalous difference Fourier maps (Schiltz *et al.*, 2003 ▸).

### 3.2. Investigation of gas-employing enzymes

*A priori*, noble gases have proved to be good mimics for mapping diffusion tunnels and labelling storage sites for reactive gases (such as oxygen, carbon dioxide, methane, nitrogen *etc.*; see Section 3.1) that are commonly used by many enzymes to function. Nevertheless, it is always preferable, whenever possible, to use a genuine non-inert gas in order to more precisely reveal the gas diffusion and binding processes, and also to induce enzymatic reactions when these gases are substrates. Indeed, enzyme–gas complexes or reaction intermediates can be trapped *in crystallo* by flash-cooling protein crystals after a certain soaking time in an appropriate pressurized pure gas atmosphere, in the same way as crystals are usually soaked in substrate-enriched solutions to trigger and freeze reaction intermediates in crystals using kinetic crystallography methods (Bourgeois & Royant, 2005 ▸). Thus, this application of pressurized gas allows the molecular mechanisms of enzymatic reactions involving the transformation of gaseous substrates to be dissected (feature 5 in Fig. 1 ▸). To this end, we designed specific cryogenic high-pressure cells to allow crystals to be exposed to oxygen, carbon dioxide and methane. Molecular oxygen is essential in many biological processes. O_2_ is a stable molecule that can be activated to perform various oxidation processes (Poulos, 2014 ▸; Romero *et al.*, 2018 ▸). For this, O_2_ binds to various proteins (such as hemoproteins) or is the substrate of enzymes such as redox enzymes (for example oxidases, oxygen reductases, oxygenases, dehydrogenases *etc.*) for which it can act as an oxidizing agent (Bui *et al.*, 2023 ▸). In contrast, O_2_ and in particular its activated forms can also produce oxidative damage to many other proteins, which is often connected to serious damage and tightly controlled by repair mechanisms. Indeed, many enzymes are oxygen-sensitive and their catalytic activities are inactivated upon exposure to oxygen (for example Fe–S cluster-containing enzymes; van der Linden *et al.*, 2022 ▸; Zacarias *et al.*, 2020 ▸; Kalms *et al.*, 2016 ▸, 2018 ▸; Imlay, 2006 ▸; Lubitz *et al.*, 2014 ▸).

There is also growing interest in the study of enzymes that are capable of processing greenhouse gases. The limitations of fossil energies accompanied by the increase in the emission of greenhouse/pollutant gases into the atmosphere (mostly carbon dioxide, but also methane) urge research into new sustainable resources such as the conversion of carbon dioxide into fuels or other valuable chemicals. For example, carbon dioxide reductase enzymes can convert carbon dioxide into carbon monoxide, formic acid or ethylene, as is typically the case for carbon monoxide dehydrogenase (CODH; Jeoung & Dobbek, 2007 ▸). In contrast, carboxylases are capable of reincorporating carbon dioxide into larger molecules, as in the case of ribulose 1,5-bisphosphate carboxylase (RuBisCO; Andersson & Backlund, 2008 ▸). Methane is another greenhouse gas, with a global warming potential 30 times higher than that of carbon dioxide, and many studies are focusing on strategies to reduce its emission into the atmosphere. This gas is the substrate and product of various methane-dependent enzymes (typically ethane monooxygenases and methyl-coenzyme M reductases). Carbon dioxide and methane pressure cells are being used to support structural studies to understand how these biological molecules process these gases and to design inhibitors against bio-methane production by methanogens.

Interestingly, for this application of high-pressure experiments (the introduction of gaseous substrates into enzymes), structure determination can often be complemented by spectroscopic data to characterize the gas–protein complexation state or the reaction step in the crystal (using, for example, the *ic*OS laboratory; von Stetten *et al.*, 2015 ▸; Royant *et al.*, 2007 ▸). Indeed, many gas-processing enzymes contain a metal centre or a cofactor that activates or processes the gas molecule (such as oxygen, carbon dioxide or methane) via a redox mechanism. The state of these reactive centres (reduced or oxidized) can be determined in the crystal, after gas derivatization (possibly at different pressure-soaking times), by UV–Vis spectroscopy and can be compared with that of the native protein. Additionally, the presence of the gas molecule in the crystal can also be confirmed by *in crystallo* Raman spectroscopy (Katona *et al.*, 2007 ▸; von Stetten *et al.*, 2015 ▸; Carpentier *et al.*, 2007 ▸).

### 3.3. Structure modifications and conformational fluctuations

For proper functioning, macromolecules need to carry out internal motions ranging from atomic vibrations and side-chain conformational fluctuations up to entire domain movements, which take place on time scales from femtoseconds to milliseconds. Crystallographic structures are in general an averaged representation of these fluctuations, which can be identified by high atomic temperature factors, side-chain conformational disorder or poor electron-density sections. Populations of conformational sub-states are governed by thermodynamics, but most of them are not observed in structures since protein motions are somewhat restricted in the crystalline environment and since most of these sub-states are observed at high energy levels and are not populated at ambient pressure and cryogenic temperature. Pressure is a thermodynamic variable which can aid in the exploration of the energy landscapes of proteins by shifting the sub-state population by a factor exp(−*P*Δ*V*/*RT*) towards those of smaller volumes (where Δ*V* is the volume variation between two sub-states, *R* is the gas constant and *T* is the temperature). Hence, high pressure (typically from 500 to 2000 bar) allows the population, in crystals, of functionally relevant protein conformations that are not observed under ambient conditions. Once populated in crystals under pressure, these new protein conformational states are cryo-trapped and can be surveyed in structures using classical crystallography (feature 6 in Fig. 1 ▸; Collins *et al.*, 2011 ▸; Barstow *et al.*, 2008 ▸, 2009 ▸; van der Linden *et al.*, 2014 ▸). Similarly, we also found that the application of pressure to some protein–ligand complexes in crystals allows a shift in the thermodynamic equilibrium towards saturation of ligand occupancies in protein binding sites, but also, in the same manner, enables the population of less stable secondary ligand-binding sites that are unpopulated at atmospheric pressure (Prangé *et al.*, 2022 ▸). This effect of pressure proves to be essential for dissecting enzymatic reaction intermediates in crystals and highlighting intermediate stages of ligand–protein binding processes. After being populated by pressurization, these sub-states are trapped by flash-cooling with the crystals still under pressure, and they remain stable for structural investigations provided that the samples are maintained at cryogenic temperatures. Pressure-induced structural modifications are difficult to predict beforehand, but often result simply in a global increase in *B* factors (van der Linden *et al.*, 2014 ▸). This unfavourable effect nevertheless allows a distinction between stable structural elements (generally highly structured elements, such as α-helices and β-strands) and flexible elements that are more sensitive to pressure (generally extended loops). In some cases, pressure also modifies the protein–protein interactions involved in crystal packing and thereby induces a change of symmetry (*i.e.* phase transitions; see Section 4.4; Prangé *et al.*, 2022 ▸).

### 3.4. Cryo-cooling protein crystals without cryo-protection

The search for effective and minimally disruptive cryo-protectants to flash-cool protein crystals while avoiding solvent icing but preserving the crystal quality (*i.e.* the diffraction resolution and mosaicity) is a tedious empirical task and crystallo­graphers often prefer to add simply glycerol to the mother liquor in order to rapidly protect their samples without further optimization. Cryo-protectants (such as glycerol) are non-inert chemical molecules that may interact with the protein, causing structural modifications or unwanted occupation (for example in the active site), which may even lead to structural misinterpretation. In the extreme case of very fragile crystals, it is sometimes impossible to find a suitable cryo-protectant that allows cryo-cooling without destroying the crystalline order. Together, these drawbacks led crystallo­graphers to search for alternative methods that allow the cryo-cooling of crystals without adding any extra chemicals, such as for instance improved cooling efficiency (Warkentin *et al.*, 2006 ▸) or a reduction of the solvent surrounding the sample (Pellegrini *et al.*, 2011 ▸). A more methodical approach is the flash-cooling of crystals using high pressure (Kim *et al.*, 2005 ▸; van der Linden *et al.*, 2014 ▸). Indeed, the phase diagram of water shows that the isobaric flash-cooling of crystals at 2000 bar (HP-cool; Fig. 2 ▸ *b*) allows the transformation of solvent water directly into high-density amorphous ice (HDA), passing rapidly through the phase of crystalline ice type II without crystallizing the water. On the contrary, at atmospheric pressure the solvent requires a cryo-protectant prior to being flash-cooled into the low-density amorphous phase (LDA), otherwise the water within the liquid is immediately converted into crystalline ice type I (LP-cool; Fig. 2 ▸ *b*). Advantageously, the high-pressure cooling method has proved to be systematically applicable to all biological crystals independent of the crystallization liquid, and the HDA vitreous matrix at high pressure better preserves the protein crystal quality than the LDA at atmospheric pressure (Kim *et al.*, 2005 ▸; van der Linden *et al.*, 2014 ▸). The limitation of the pressure-cooling method is that it requires specialized high-pressure instruments that are necessarily run by expert operators and hence are preferably installed at large facilities.

## 4. Examples and discussion

### 4.1. Studies of tunnel networks in oxygen-tolerant hydrogenases

**Figure 5:**
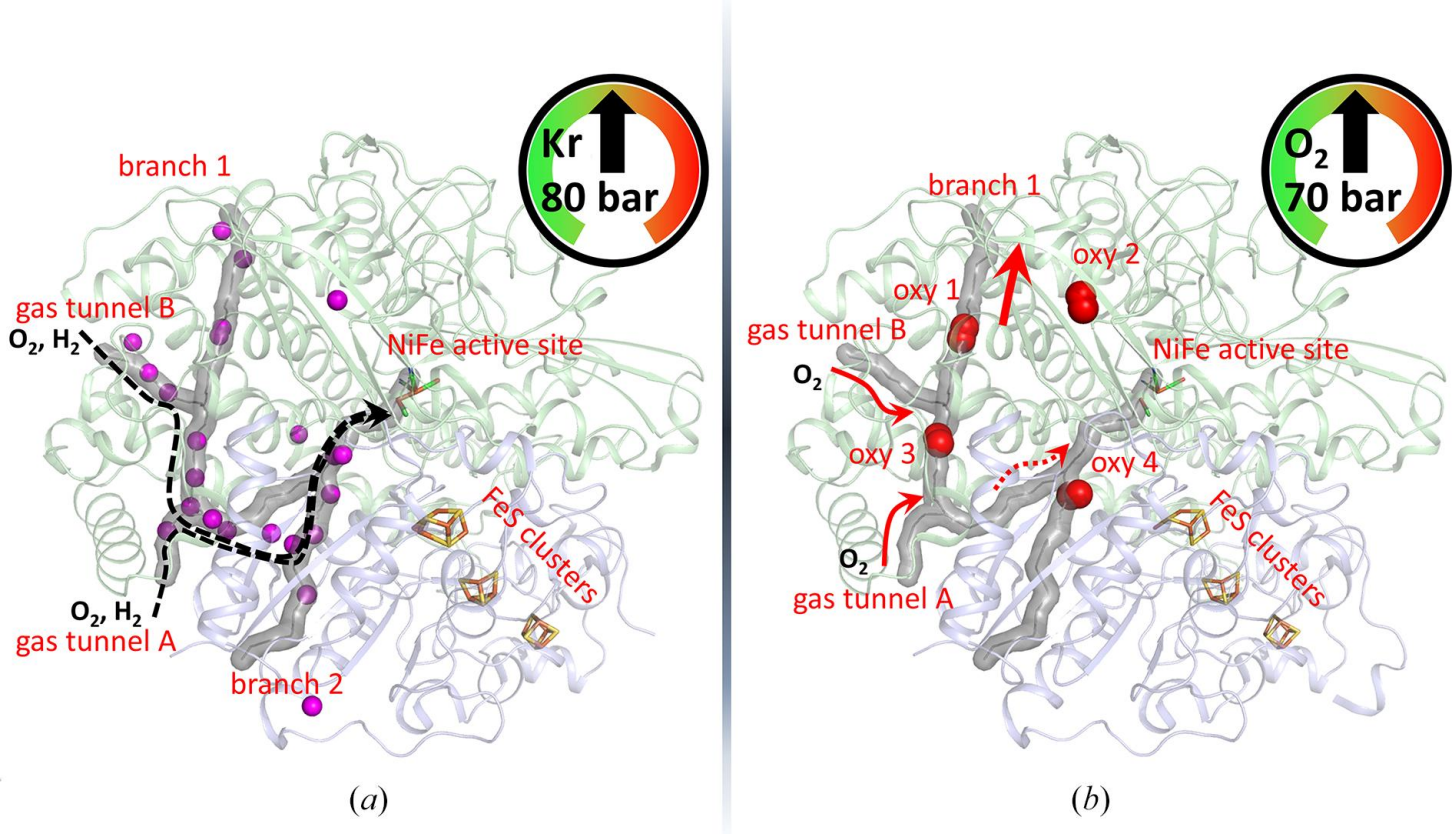
Gas-tunnel network in *R. eutropha* [NiFe]-hydrogenase. (*a*) The structure of a krypton derivative of ReMBH reveals the pathway for both H_2_ and O_2_ (PDB entry 5d51). The 19 Kr atoms are represented as magenta spheres. The tunnel network is mapped (using *Caver*) as a grey surface and comprises two main tunnels and two branches. (*b*) Structure of the O_2_ derivative (PDB entry 5mdl) reveals the locations of four oxygen molecules; molecular dynamics suggest that O_2_ is preferentially redirected (red arrows) away from the NiFe active site.

Hydrogenases are prototypical examples of gas tunnel-containing enzymes. They are metallo-enzymes that are capable of catalyzing the reversible oxidation of molecular hydrogen into two protons and two electrons. These proteins protect their active site deeply buried within their core, and hydrogen molecules in the solvent are required to diffuse to the catalytic centre via hydrophobic tunnels for the enzymatic reaction to proceed. Two examples of hydrogenases have been investigated at the HPMX laboratory: *Desulfovibrio vulgaris* [NiFeSe]-hydrogenase (Zacarias *et al.*, 2020 ▸) and *Ralstonia eutropha* [NiFe]-hydrogenase (ReMBH; Kalms *et al.*, 2016 ▸, 2018 ▸). These enzymes are both described to be more tolerant to atmospheric oxygen than most other ordinary hydrogenases, and are thus of interest for potential biotechnological applications. Since the pathways for gas molecules (H_2_ and O_2_) in ReMBH were largely unknown, gas derivatives of ReMBH crystals using high pressure in pure krypton and oxygen atmospheres (80 and 70 bar, respectively) were produced in order to visualize the hydrophobic channels and to track the presence or reactivity of oxygen molecules. One of the essential biochemical questions was to understand the possible relationship between the internal architecture of ReMBH and its oxygen tolerance. The interpretation of an anomalous difference Fourier map revealed a continuous series of 19 krypton sites (collected at the Kr edge on beamline ID23-1; Nurizzo *et al.*, 2006 ▸) that allowed precise mapping of the internal protein architecture of ReMBH (Fig. 5 ▸ *a*). The structure analysis demonstrated that the hydrogen substrate and inhibitory oxygen diffuse indistinguishably through the same routes. A comparison of different hydrogenases showed that oxygen-tolerant enzymes contain on average shorter and half as many hydrophobic gas channels than oxygen-sensitive enzymes, which probably contribute to a more stringent control of the flow of gas molecules and improved H_2_ selectivity. Based on the O_2_-derivative structures (Fig. 5 ▸ *b*), diffraction data collected on beamline ID30B (McCarthy *et al.*, 2018 ▸; Mueller-Dieckmann *et al.*, 2015 ▸) and molecular-dynamics simulations demonstrated that the ReMBH channel system has a tendency to favour the direct diffusion of H_2_ towards the active site, while O_2_ is redirected towards a secondary branch system to protect the enzyme from inactivation. These high-pressure crystallographic studies (Kalms *et al.*, 2016 ▸, 2018 ▸) provide the structural determinants that confer the significant tolerance of ReMBH towards atmospheric oxygen. On this basis, improved hydrogenases could be designed by modifying the tunnel characteristics through site-directed amino-acid modifications.

### 4.2. Tracing of non-predicted tunnels in proteins

**Figure 6:**
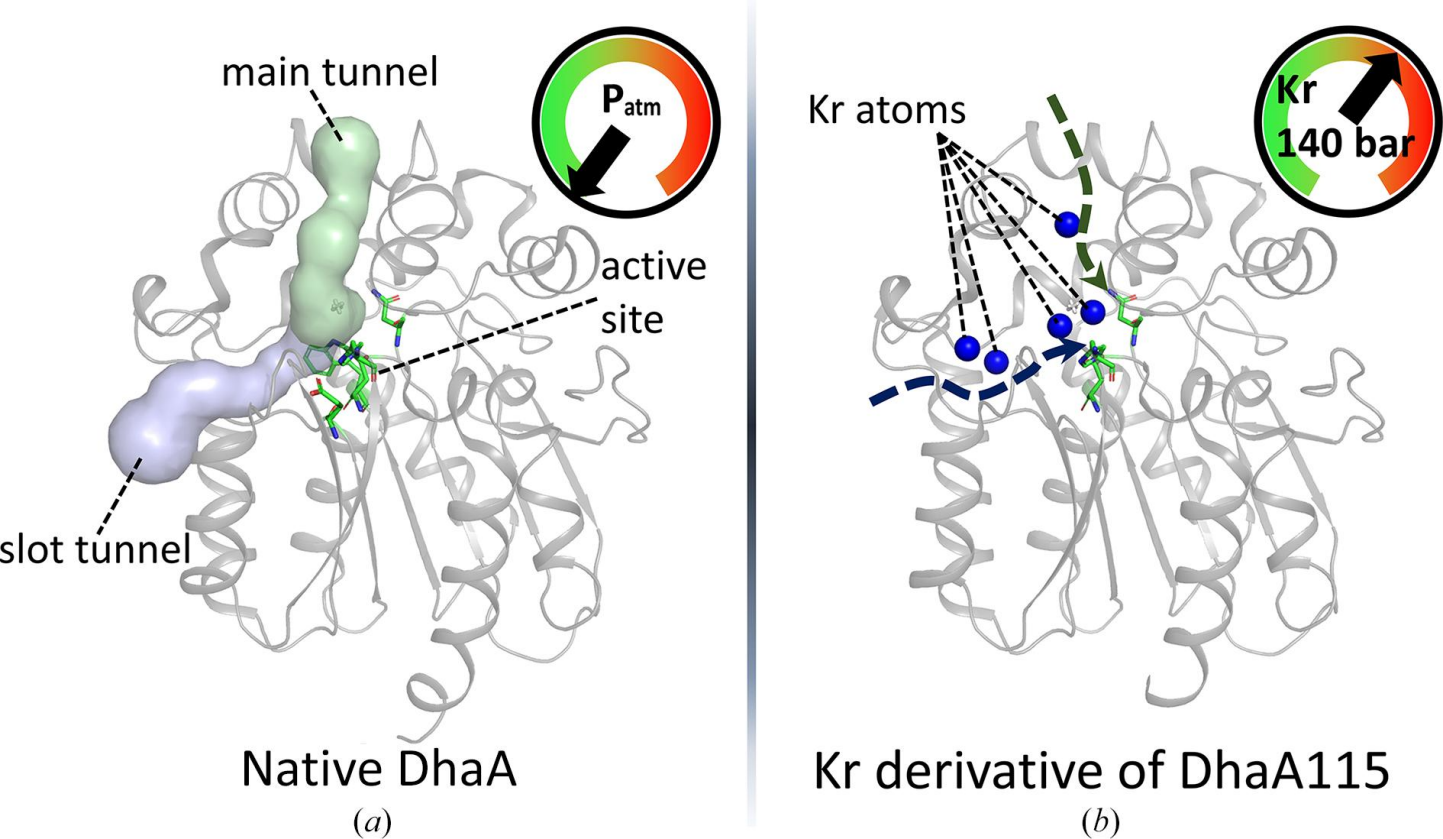
Access to the active site in the haloalkane dehalogenase DhaA. (*a*) Structure of native DhaA; the active site is connected to the solvent by a main tunnel and a slot tunnel (PDB entry 4hgz). (*b*) Structure of the krypton derivative of the mutant DhaA115 (PDB entry 6sp8). Although the accesses to the active site of DhaA115 are closed at atmospheric pressure (*Caver* calculations), the krypton derivative (pressurized at 140 bar) reveals a series of Kr atoms demonstrating the effective accessibility of the active site.

An original investigation carried out at the HPMX laboratory showed that high-pressure krypton labelling of protein crystals (Markova *et al.*, 2020 ▸) allows protein tunnels to be revealed in proteins that are not predicted by dedicated computational programs (such as *Caver* or *MOLE*2; Sehnal *et al.*, 2013 ▸; Chovancova *et al.*, 2012 ▸). DhaA is a haloalkane dehalogenase from *Rhodococcus rhodochrous* that catalyzes the cleavage of carbon–halogen bonds in carbon halide compounds and can potentially serve as a bioremediator. The buried active site of DhaA is connected to the solvent by a main tunnel and a slot tunnel that are both essential determinants of the catalytic activity of the enzyme (DhaA native; Fig. 6 ▸ *a*). DhaA115 is a mutant of DhaA designed *in silico* to be highly thermostable and has demonstrated an optimal enzymatic activity (*T*_opt_) of 65°C *in vitro* (compared with a *T*_opt_ of 45°C for the wild type), with interesting properties for potential industrial applications. On the basis of structural studies, computation of active-site access routes in DhaA115 using *Caver* (Chovancova *et al.*, 2012 ▸) suggests that both the main tunnel and the slot tunnel are blocked by some of the mutated residues, which was a counterintuitive observation since the mutated enzyme still displayed strong activity. The Kr atoms observed throughout the apparently occluded tunnels (main and slot) in the structures of this derivative demonstrate that the DhaA115 enzyme (Fig. 6 ▸ *b*) is still highly permeable and the active site is accessible to both substrates and products (PDB entries 6sp5 and 6sp8; data collection on beamline ID23-1). The explanation for this surprising observation lies in the fact that the permeability certainly increases with temperature, enabling the enzyme to achieve optimum activity at higher temperatures (*T*_opt_ = 65°C). This thermal increase in permeability is well mimicked by pressure, but could not be simulated by the tunnel-calculation programs. This result provides a structural basis for the design of new enzymes with improved stability (Markova *et al.*, 2020 ▸).

### 4.3. Probing the surface of membrane proteins using pressurized argon and krypton

**Figure 7:**
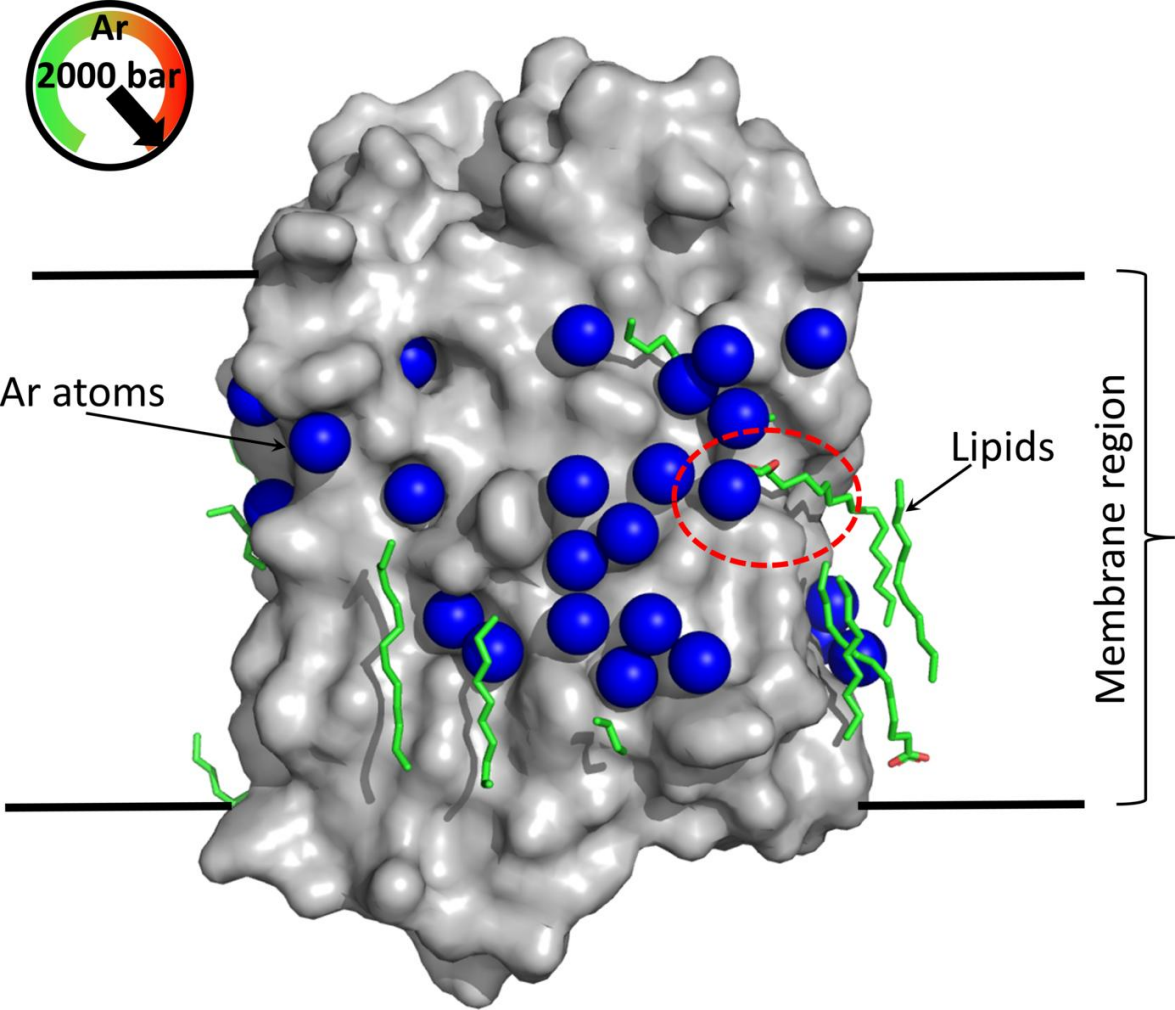
Structure of an argon derivative of bacteriorhodopsin (from PDB entry 7q38). The surface of the protein (with its excavations) is represented in grey. The structure contains 47 Ar atoms (blue spheres) identified in an anomalous difference Fourier map. Lipids are represented as green sticks. Argons compete and displace some lipids from their native positions (red circles).The hydrophobic region is delimited by horizontal lines.

A recent study has demonstrated the strong potential of the use of pressurized noble gases to explore and investigate, *in crystallo*, the functioning of membrane proteins (MP) with their lipids (Melnikov *et al.*, 2022 ▸). To identify the general tendency of the action of pressurized noble gases on MP–lipid systems, this study investigated the effects of noble gases on three different MPs with well characterized structures: a bacteriorhodopsin (BR), a proton-pump rhodopsin (MAR) and a sodium pump (KR2). Noble-gas derivatives of these MP crystals were produced by the soak-and-freeze method available at the HPMX laboratory using high-pressure atmospheres of pure krypton and argon gas at 130 and 2000 bar, respectively. The krypton and argon gas atoms, even at low occupancies, were easily located in the structures using the anomalous scattering contribution at the Kr edge (0.866 Å) or at longer wavelength (1.85 Å) for argon (data were collected on beamline ID23-1; Nurizzo *et al.*, 2006 ▸). This study clearly revealed that noble-gas atoms have a high tendency to bind to the hydrophobic surface of MPs, and thus were observed to occupy pockets on the outer hydrophobic surface (Fig. 7 ▸). More interestingly, these noble-gas atoms appear to compete with lipids, which have also their hydrophobic acyl chains anchored in grooves on the protein hydrophobic surface region. Some of these lipid molecules were observed to be displaced from their native positions by Ar atoms. This effect of noble gases on MPs, which seems to be harmless at first sight, nevertheless has important consequences since the lipid environment is among the main determinants of MP functionality and specific structural lipids are strictly necessary to allow MPs to perform their proper functions. Complementary molecular-dynamics simulation analysis confirmed these observations and demonstrated that MPs undergo a reduction of their dynamics upon binding noble-gas atoms, which *de facto* affects their functional processes. Interestingly, since the noble-gas perturbation mechanism should be transposable to neuronal MPs, which are anaesthetic targets, this result has been proposed to be a common feature of the action of anaesthetics (Melnikov *et al.*, 2022 ▸).

### 4.4. Structure modifications induced by high pressure

**Figure 8:**
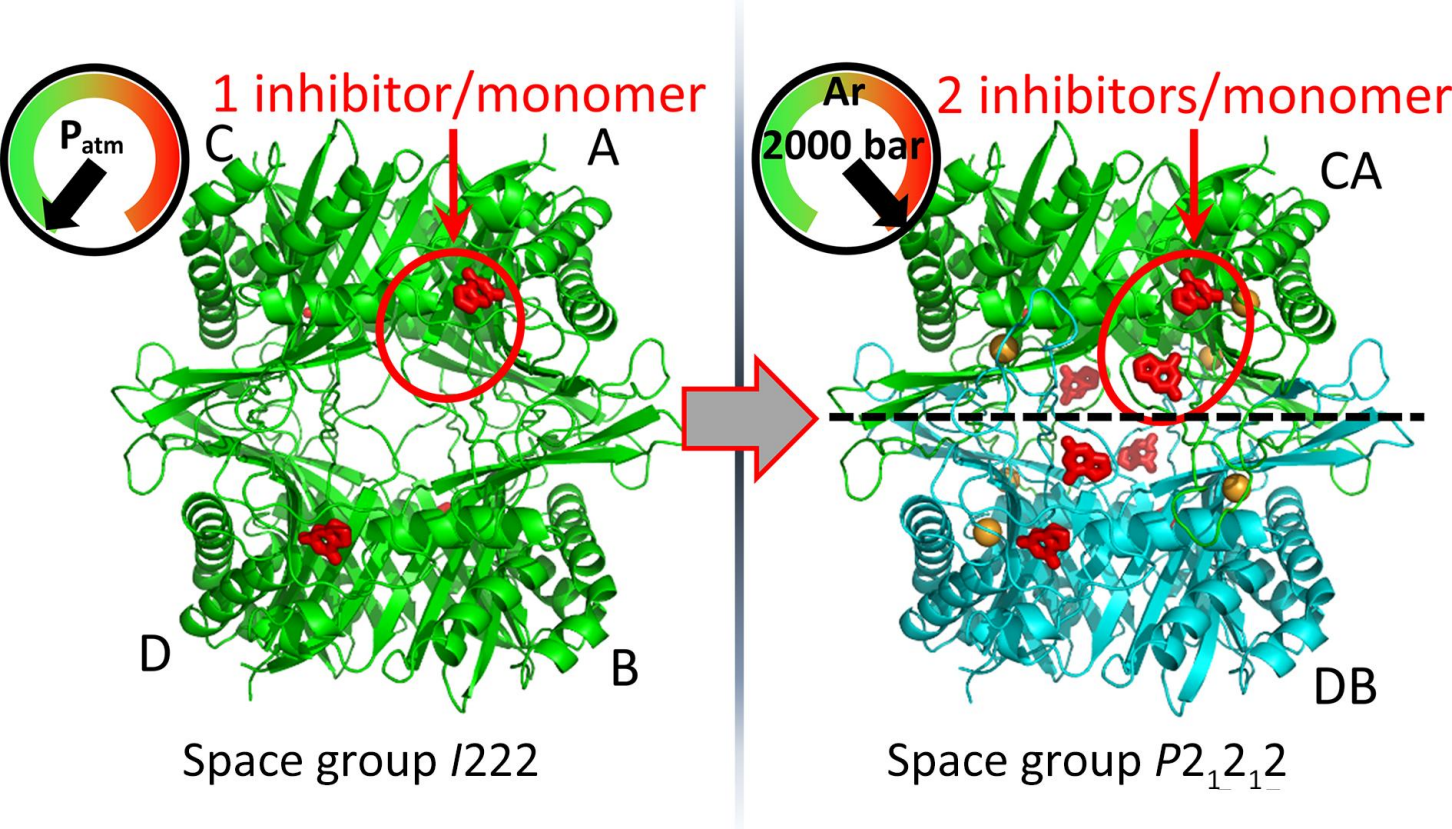
Pressure induces structural change in UOX crystals. A phase transition from *I*222 to *P*2_1_2_1_2 occurs at 600 bar, during which the tetramer switches from an α_4_ to an (αβ)_2_ assembly (shown in green and in green/cyan, respectively). A second AZA inhibitor is observed above 1500 bar (PDB entry 6ia9).

A recent structural study involving urate oxidase (UOX) carried out at the HPMX laboratory shows that pressure can be a profitable tool to explore functional structural changes in proteins (Prangé *et al.*, 2022 ▸). UOX is an enzyme that catalyzes the oxidation of uric acid and is used as treatment for acute hyperuricemia. In the presence of the inhibitor 8-azaxanthine (AZA), the protein crystallizes in space group *I*222 in complex with a single molecule of AZA locked in the active site (Fig. 8 ▸ *a*) and with a single monomer per asymmetric unit (Gabison *et al.*, 2008 ▸, 2010 ▸). A systematic structural study of urate oxidase was carried out using different increasing pressures of argon from atmospheric pressure up to 2000 bar. Interestingly, at 600 bar the UOX crystals underwent a phase transition from space group *I*222 to *P*2_1_2_1_2. At the structural level, the modifications induced by pressure are weak and involve only a few short regions that make contact between the UOX monomer mates, but they are sufficient to induce loss of the 222 symmetry in the α_4_ tetramer, thus leading to a (αβ)_2_ tetrameric assembly. Pressure-induced disruption of the symmetry at the *AC*–*BD* interface was interpreted as an early stage of the pressure-induced dissociation of the homotetrameric organization into two homodimers (Fig. 8 ▸). The pressure also modifies the structural state at the active site. Although the atmospheric pressure structure displays only a single inhibitor molecule locked in the active site, surprisingly at higher pressures (1500 bar and above) a second inhibitor molecule is additionally stabilized at the entrance to the hydrophilic channel connecting the solvent to the active site (Fig. 8 ▸). Hence, high pressure shifts the thermodynamic equilibrium of the UOX–AZA complex towards saturation of ligand occupancies and reveals a functional secondary binding site with lower stability, indicating how the inhibitors and possibly also the genuine substrate (uric acid) diffuse to the active site (via an intermediate binding site) and revealing part of the function of the enzyme.

## 5. Conclusion and perspectives

Since its opening in 2015, the HPMX laboratory has enabled ESRF MX users to study the interactions and reactivities of gases with crystallized macromolecules. The soak-and-freeze method allows the proper cryo-trapping of gas–protein complexes during pressurization at higher pressures and in a more secure manner than previous methods. From a scientific point of view, the method allows the population of low-affinity gas-binding sites using volatile gases; studies of reactivities *in crystallo* using non-inert gases (oxygen, carbon dioxide and methane) can thus be envisaged, opening up the field of scientific perspectives. Finally, this method makes it possible to design a series of different gas pressure cells based on the same concept, sharing a common instrumentation and installed in a unique facility (the HPMX) accessible to the whole MX community. Over time, the laboratory has increased its capabilities, offering new cryogenic pressure cells for cooling macromolecular crystals in the presence of pure gas atmospheres (helium, argon, krypton, xenon, oxygen, carbon dioxide and methane are currently available) in order to meet user needs.

The laboratory has contributed to various original scientific achievements, as illustrated above, through the mapping of internal protein architecture, the reactivity of non-inert gases with enzymes, noble-gas derivatization to solve the phase problem for new structures, the exploration of conformational changes and flash-cooling without adding cryo-protection. As shown by the fact that the majority of krypton and argon derivatives deposited in the Protein Data Bank to date (June 2023) were produced at the HPMX laboratory, the soak-and-freeze method has proven its effectiveness in introducing and cryo-trapping highly volatile gases under pressure in macromolecular crystals.

There are numerous biologically interesting macromolecules that are dependent on other light molecular gases for which the macromolecule–gas interactions (reaction, complexation, transport *etc.*) still need to be studied. Examples of these are hemoproteins that bind carbon monoxide (CO) and nitric oxide (NO), nitrogen dioxide reductases that convert NO_2_ into NO, sulfide:quinone oxidoreductases that convert H_2_S to persulfide, ammonia (NH_3_) transporters (see Table 1 ▸) *etc.* Moreover, these molecular gases also intrinsically have many important biological functions; for example, they can act as signalling molecules that trigger biochemical changes in cells (for example, NO is a signalling molecule in the cardiovascular system). However, the low number of deposited structures containing gas molecules shows that the high volatility and/or the low affinity of these gases for macromolecules make it difficult to have these gaseous molecules present in crystals in sufficiently high quantities that they can be clearly interpreted in electron-density maps. Furthermore, a PDB search for 12 common gas atoms and molecules (Ar, Kr, Xe, O_2_, CO, CO_2_, N_2_, NH_3_, NO, NO_2_, N_2_O and H_2_S) resulted in 1718 hits, which represents only 0.83% of all deposited structures, clearly indicating an underrepresentation of these gases in macromolecular structures. Future developments of novel pressure cells for CO, NO, N_2_O, NO_2_, NH_3_ and H_2_S are of very great interest to fill the current technological gap and aid in studying the above-mentioned gas-dependent proteins or biological functions. However, these gases are hazardous and thermodynamically difficult to handle, and require several technological and safety issues to be overcome.

*Note added in proof.* The laboratory ‘High-pressure freezing’ and the method ‘soak-and-freeze’ names are used for historical reasons only. However, ‘cryo-cooling’ is the appropriate term (as we try to avoid a change of phase), and is the one we used in the article to describe the physical sample treatment processes.

## Figures and Tables

**Table 1:** Number of PDB depositions of protein structures containing the most common biologically active gases For noble gases, 17 of the 31 krypton derivatives and 16 of the 21 argon derivatives deposited in the PDB (as of June 2023) were produced at the HPMX laboratory.

Gas (acronym in the PDB, name)	No. of PDB depositions	Protein functions or gas properties
O_2_ (OXY, oxygen; PER, peroxide ion)	559	Oxidoreductases: oxygenases, oxidases
CO (CMO, carbon monoxide)	410	Hemoproteins, gas storage and transport
NO (NO, nitric oxide)	177	Biological mediator
CO_2_ (CO_2_, carbon dioxide)	126	Carbonic anhydrases, carboxylases, CO_2_ reductases
N_2_O (N_2_O, nitrous oxide)	17	Nitrous oxide reductases
NH_3_ (NH_3_, ammonia)	25	Ammonia transporters
H_2_S (H_2_S, hydrogen sulfide)	38	Protein sulfhydration
NO_2_ (NO_2_, nitrogen dioxide)	161	Nitrite reductases
N_2_ (HDZ, nitrogen)	8	Nitrogenases
Xe (Xe, xenon)	145	Chemically inert, biological and medical properties: analgesia, anaesthesia, neuroprotection. Methodological applications.
Kr (Kr, krypton)	31	
Ar (Ar, argon)	21	

**Table 2:** Physical properties of the different gases used at the HPMX laboratory Missing values (—) are either inaccessible or not used. Xenon and nitrogen pressure systems are available but these gases are not yet in demand at the HPMX.

Gas	He	Ar	Kr	Xe	O_2_	CO_2_	CH_4_	N_2_
Polar property	Nonpolar, inert				Quadrupole moment, reactive			
Polarizability (Å^3^)	0.2	1.6	2.5	4.0	1.6	2.5	2.4	1.7
X-ray beam energy (keV)	—	6	14.3	6	—	—	—	—
Anomalous *f*′′ (e^−^)	—	1.5	3.8	11.6	—	—	—	—
Solubility (m*M* bar^−1^)	0.4	1.4	2.5	4.3	1.3	35	1.4	0.6
Kinetic diameter (Å)	2.6	3.4	3.6	4.0	3.5	3.3	3.8	3.6
Icing temperature (K)	2.2	84	116	161	54	195	91	63
Maximum pressure at HPMX (bar)	2000	2000	500	—	70	57	50	—
